# T212. THE INTRINSIC ORGANIZATION OF SYMPTOMS MARKS TRANSITION FROM HIGH-RISK STATE TO EARLY PSYCHOSIS: A PHENOMENOLOGICAL CONNECTIVITY STUDY

**DOI:** 10.1093/schbul/sby016.488

**Published:** 2018-04-01

**Authors:** Lena Palaniyappan, Tushar Das, Fabienne Harrisberger, Undine E Lang, Anita Riecher-Rössler, André Schmidt, Stefan Borgwardt

**Affiliations:** 1 University of Western Ontario; 2 University of Psychiatric Clinics Basel (UPK)

## Abstract

**Background:**

In psychiatric practice, when symptoms “come together” we call the resulting construct as a diagnosis. We believe that there is a disease process that binds together, enabling co-occurrence of varied symptoms. We use either diagnostic or syndromic labels to describe this construct (e.g. positive syndrome, negative syndrome, schizophrenia, at-risk mental state). An emerging idea, promoted by network theorists, is that symptoms may relate by their own intrinsic nature, with no external constructs bringing them together e.g. paranoia leads to social withdrawal, loss of appetite leads to loss of weight etc. This intrinsic organisation of symptom relationships can be studied using network models by applying graph theory to symptom data.

**Methods:**

We recruited 63 subjects with at-risk mental state [on the basis of Melbourne PACE criteria] but no transition (ARMS-NT), 16 that later developed psychosis (ARMS-T) and 38 drug-naïve patients with first-episode psychosis (FEP) from Basel, Switzerland. Symptoms were measured using Brief Psychiatric Rating Scale. Clinical symptoms can be construed as a system of individual elements (24 nodes) and their relationship (24x23 possible edges) within a group. We estimate each individual’s contribution to the intrinsic organisation of symptoms using a jack-knife bias estimation procedure. Bias values for each pair of symptoms in an individual subject quantified the contribution of that subject to the overall within-group relationship for that symptom pair. Higher values meant greater relationship between the two given nodes in that subject, relative to the rest of the group. We then used Graph Analysis Toolbox, with a range of binarization thresholds based on cost-density of connectivity to extract adjacency matrices.

**Results:**

None of the 24 individual symptoms of BPRS significantly differentiated ARMS-NT from ARMS-T, though a number of symptoms (suspiciousness, hallucinations, disorganisation, motor retardation, hostility and suicidality) showed a gradient of FEP>ARMS-T>ARMS-NT (F test, FDR corrected p<0.05). The small-worldness (F=4.8, p=0.01) and the clustering coefficient (F=10.9, p<0.001) and modularity (F=10.9, p<0.001) of the symptom networks were notably different among the 3 groups, with a gradient of FEP>ARMS-T>ARMS-NT (except for modularity where FEP=ARMS-T). Post-hoc tests revealed significantly high clustering (Hedges’s g = 0.60, p<0.05) and high modular organisation (Hedges’s g = 0.81, p<0.01) of symptoms in ARMS-T compared to ARMS-NT. There were no differences between ARMS-T and FEP groups. In both ARMS-T and FEP groups, anxiety was the most central symptom. In addition to anxiety, the FEP group also had unusual thought content emerging as a central feature.

**Discussion:**

To our knowledge, this is the first study to investigate the intrinsic phenomenological connectivity and its relevance to psychosis in the clinical high-risk population. Risk of transition to psychosis relates to the consolidation of relationship among symptoms (clustering and modularity), but appears unrelated to the severity of symptoms per se. First episode of psychosis could be thought of as a state of high modular clustering among otherwise sparsely connected symptoms. Incongruent clustering (e.g. blunting with anxiety) is reminiscent of Bleuler’s concept of ambivalence being a fundamental feature of psychosis. Deconsolidation of symptom clustering could be the key to prevent transition to frank psychosis in high-risk individuals. Reducing the bridging symptoms (esp. anxiety) could weaken the clinical core of a psychotic episode, complementing the pharmacological approaches of reducing dopamine transmission.

